# Effect of Computer Debriefing on Acquisition and Retention of Learning After Screen-Based Simulation of Neonatal Resuscitation: Randomized Controlled Trial

**DOI:** 10.2196/18633

**Published:** 2020-08-11

**Authors:** Daphne Michelet, Jessy Barre, Jennifer Truchot, Marie-Aude Piot, Philippe Cabon, Antoine Tesniere

**Affiliations:** 1 Ilumens Platform of Simulation in Healthcare Université de Paris Paris France; 2 Department of Anesthesia and Intensive Care American Memorial Hospital Centre Hospitalier Universitaire de Reims Reims France; 3 Emergency Department Lariboisière University Hospital Paris France; 4 Psychiatry Department Monsouris Mutualiste Institute Paris France; 5 Laboratoire Adaptation Travail Individu Université de Paris Boulogne-Billancourt France; 6 Department of Anesthesia and Intensive Care Georges Pompidou European Hospital Paris France

**Keywords:** screen-based simulation, debriefing, neonatal resuscitation, simulation, medical education, midwifery, neonatal

## Abstract

**Background:**

Debriefing is key in a simulation learning process.

**Objective:**

This study focuses on the impact of computer debriefing on learning acquisition and retention after a screen-based simulation training on neonatal resuscitation designed for midwifery students.

**Methods:**

Midwifery students participated in 2 screen-based simulation sessions, separated by 2 months, session 1 and session 2. They were randomized in 2 groups. Participants of the debriefing group underwent a computer debriefing focusing on technical skills and nontechnical skills at the end of each scenario, while the control group received no debriefing. In session 1, students participated in 2 scenarios of screen-based simulation on neonatal resuscitation. During session 2, the students participated in a third scenario. The 3 scenarios had an increasing level of difficulty, with the first representing the baseline level. Assessments included a knowledge questionnaire on neonatal resuscitation, a self-efficacy rating, and expert evaluation of technical skills as per the Neonatal Resuscitation Performance Evaluation (NRPE) score and of nontechnical skills as per the Anaesthetists’ Non-Technical Skills (ANTS) system. We compared the results of the groups using the Mann-Whitney U test.

**Results:**

A total of 28 midwifery students participated in the study. The participants from the debriefing group reached higher ANTS scores than those from the control group during session 1 (13.25 vs 9; U=47.5; *P*=.02). Their scores remained higher, without statistical difference during session 2 (10 vs 7.75; *P*=.08). The debriefing group had higher self-efficacy ratings at session 2 (3 vs 2; U=52; *P*=.02). When comparing the knowledge questionnaires, the significant baseline difference (13 for debriefing group vs 14.5 for control group, *P*=.05) disappeared at the end of session 1 and in session 2. No difference was found for the assessment of technical skills between the groups or between sessions.

**Conclusions:**

Computer debriefing seems to improve nontechnical skills, self-efficacy, and knowledge when compared to the absence of debriefing during a screen-based simulation. This study confirms the importance of debriefing after screen-based simulation.

**Trial Registration:**

ClinicalTrials.gov NCT03844009; https://clinicaltrials.gov/ct2/show/NCT03844009

## Introduction

Neonatal resuscitation requires training. Almost 10% of newborns and 80% of preterm newborns weighing less than 1500 g will undergo resuscitation at birth, and the quality of care provided during the first minute of life is directly linked to the outcome [1-3]. Theoretical knowledge from current guidelines [4] is essential to ensure optimal neonatal resuscitation. Several technical skills, such as bag-mask ventilation, endotracheal intubation, or umbilical catheter placement, and nontechnical skills, such as situation awareness, decision making, communication, and teamwork [4,5] are also required to ensure safety and efficacy.

Since 2011, the Neonatal Resuscitation Program (NRP) developed by the American Academy of Pediatrics includes simulation-based training. The implementation of NRP led to a decrease in neonatal and perinatal mortality [6]. Simulation training increases the trainees’ self-conﬁdence [7], knowledge [2], and technical skills [8] and improves team behavior [9]. Simulation training has many advantages such as the possibility to practice procedures without any risk for the patient and for trainees to commit errors and learn from those errors, through the repetition of different scenarios [9].

In recent years, screen-based simulation has become increasingly prevalent. They show many advantages such as better affordability than high-fidelity simulation [10], transportable, and autonomous (no need for an instructor). Screen-based simulation appears to be a valid tool in simulation-based education for health professionals, ensuring the same learning efficacy than traditional learning methods [11,12]. Indeed, recently, the development of computer sciences allowed the creation of more realistic medical simulators to improve knowledge and acquire nontechnical skills, know-how, and technical gestures [12,13]. A screen-based simulator (NRP eSim) designed by Laerdal Medical in collaboration with the American Academy of Pediatrics is even included in the NRP program as 1 of the 6 educational components of the NRP 7th edition curriculum [14].

Debriefing is inseparable from simulation. It has been shown to improve professional practice and clinical skills [15-18]. Debriefing represents a discussion between 2 or more individuals during which, aspects of a performance are explored and analyzed with the aim of gaining insights that impact the quality of future clinical practice [19]. Various efficient debriefing methods exist: postsimulation debriefing, in-simulation debriefing, verbal instructor debriefing, video-assisted instructor debriefing, self-debriefing, and multimedia debriefing (a computer text presentation with audio voice-over and videos) [15,17]. For example, Boet et al [20] showed that a self-debriefing (formative self-assessment aiming to provide feedback, allowing students to reflect on their performance and subsequently improve their skills) was as effective as traditional debriefing by an instructor. As part of simulation-based education, screen-based simulation must provide debriefing. These simulators “can easily include tools and modules of various kinds to collect data transparently during play. The data can then be processed to provide material for feedback during play, as in-game debriefing, and also as part of the end-of-game debriefing” [21]. For example, after the NRP eSim training, students received automated feedback for self-reflection. This feedback highlighted good performances achieved during the experience, the procedures that needed to be improved, and the missed procedures. The feedback represents what we refer to as “computer debriefing,” often delivered after a screen-based simulation in order to stay in a virtual environment with no need of an instructor [22]. However, few evaluations of the impact of computer debriefing on acquisition and retention of learning exist.

Retention of learning has been studied extensively after different simulation training in health sciences [23] and neonatal resuscitation [2,24,25]. However, the mean retention time of learning after simulation training and the optimal time interval between two formations remain debated [26,27]. The role of debriefing on retention of learning was already highlighted in some high-fidelity simulation studies [15,17], but it has not been studied in the context of screen-based simulation.

The objective of this study is to evaluate the impact of a computer debriefing after a screen-based simulation session compared to no debriefing in a virtual environment with no instructor. Our endpoints are acquisition of knowledge and skills and their retention after 2 months. We hypothesized that the debriefing group would yield better scores in different evaluations (knowledge, technical skills, nontechnical skills, and self-efficacy) as compared to the control group.

## Methods

This randomized controlled simulation study was performed from November 2018 to January 2019 at L’école de Sages-Femmes de Baudelocque, a midwifery school of the Université de Paris. It was approved by the CERAR (Comité Ethique sur la Recherche en Anesthésie Réanimation) (IRB 00010254-2017-008). All students signed an informed written consent. The study was registered at ClinicalTrials.gov (NCT03844009).

### Participants

Volunteer participants were recruited from among fourth-year students of L’école de Sages-Femmes de Baudelocque in Paris. They all followed the same curriculum on neonatal resuscitation, corresponding to only 1 academic course. No sample size calculation was performed for this research; a convenience sample was used. We included all 28 volunteers of the fourth-year class of 35 students.

### Screen-Based Simulation

**Figure 1:**
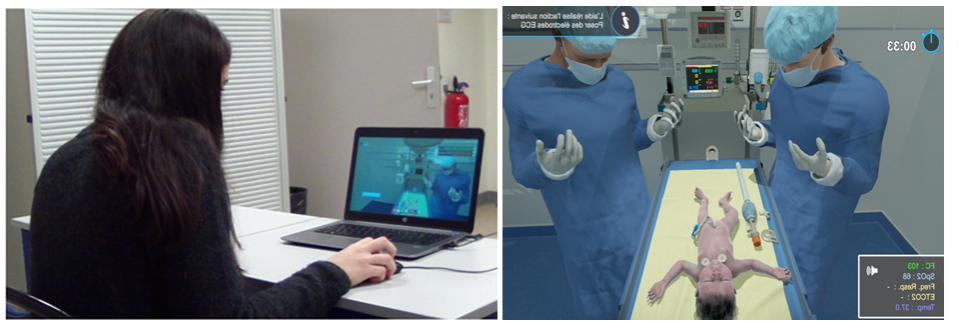
Participant during a scenario on the left and screenshot of the interface and virtual environment of Périnatsims screen-based simulation on the right.

The screen-based simulation—Périnatsims—was designed by Medusims. It features the virtual environment of a delivery room in 3D with a newborn installed on a neonatal resuscitation table (Figure 1). The simulation used a point-and-click interface with a first-person point of view. In this digital simulator, learners could either be midwife, anesthetist, or pediatrician, although all the participants of this study were midwifery students. Throughout the scenario, the learner can call a pediatrician for help. Many scenarios were available with different difficulty levels (eg, preterm birth, emergency cesarean under general anesthesia, and abruptio placentae).

### Design

**Figure 2:**
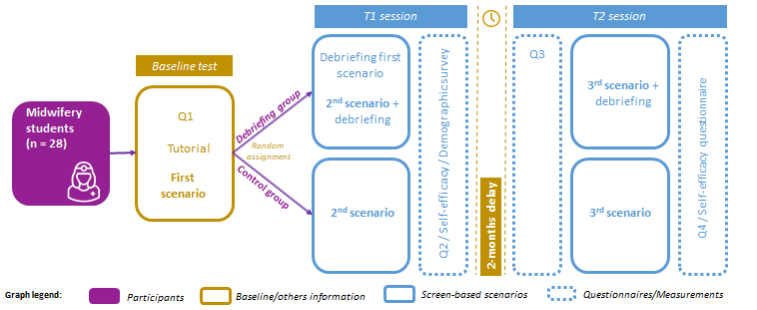
Experiment design.

Each participant performed individually on a laptop during 2 screen-based simulation sessions: session 1 in November 2018 and session 2 in January 2019 (Figure 1). The study design is summarized in Figure 2.

Session 1 started with a knowledge questionnaire (Q1, baseline), followed by the briefing, consisting of a 15-minute tutorial to explain different possible actions in the screen-based simulation. The participant performed the first scenario (low difficulty level) considered as the baseline level for knowledge and skills. It was followed by a second scenario (medium difficulty level). At the end of session 1, each participant again filled out the knowledge questionnaire (Q2) and a demographic survey, including a self-efficacy question.

Session 2 was conducted after 2 months. The simulation started with the same knowledge questionnaire (Q3) and tutorial, followed by a third scenario (high difficulty level). At the end of session 2, a last knowledge questionnaire (Q4) and the self-efficacy question were administered.

All 3 scenarios were identical and in the same order of increasing difficulty for every participant. The potential exposure of each participant to a real case (or training) of neonatal resuscitation during the 2 months delay was controlled and monitored.

**Figure 3:**
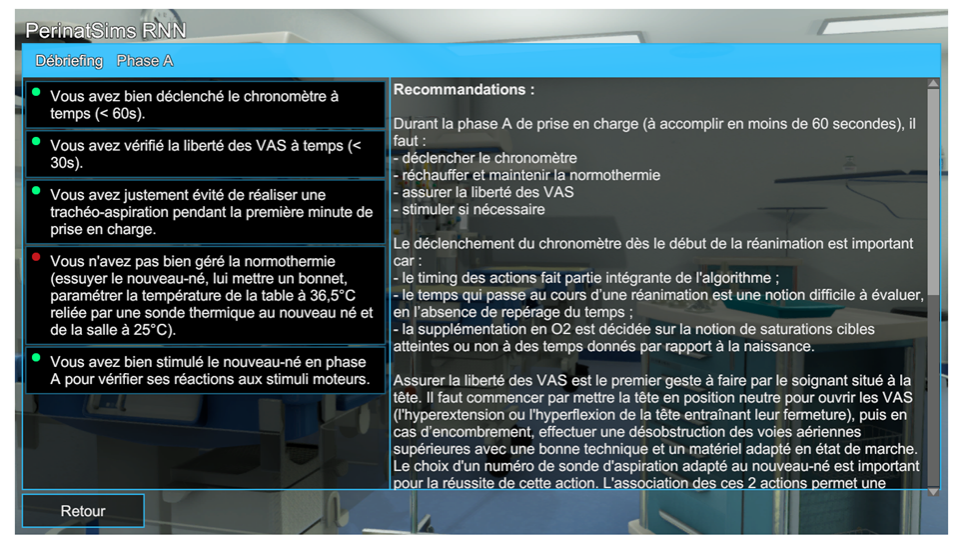
Computer debriefing of technical skills.

Participants were randomized in 2 groups: debriefing group and control group. At the end of each scenario, participants from the debriefing group accessed a computer debriefing on technical and nontechnical skills. Technical skills assessment, based on the recommendations of International Liaison Committee on Resuscitation (ILCOR), was presented with a color code: green (well-performed action), orange (partially-performed action), and red (absent or wrong action) (Figure 3).

**Figure 4:**
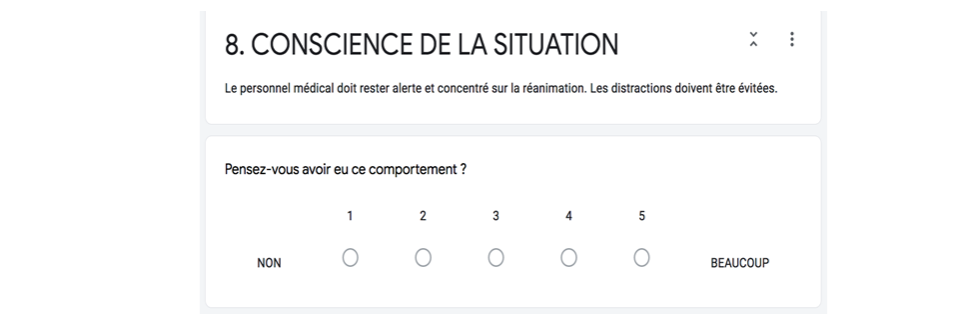
Self-debriefing of nontechnical skills.

Debriefing of the nontechnical skills was a self-debriefing. Each nontechnical skill involved in the neonatal resuscitation [3-5,28] was explained in one sentence, and then the learner self-rated their proficiency on a scale of 1 to 5, as shown in Figure 4. In this example, the nontechnical skill of situational awareness is explained as, “Medical staff have to stay alert and focus on the resuscitation. Distractions must be avoided.” The following question is, “Do you think you had this behavior?”

Participants from the control group had no debriefing until the end of the second session and the completion of every questionnaire. The sessions were recorded using a camera with participants’ written consent.

### Outcomes

#### Comparison of Knowledge Acquisition and Retention

Knowledge was assessed using validated questionnaires (25 questions with single or multiple choices) based on the ILCOR recommendations [29]: Q1 at the beginning of session 1, Q2 at the end of session 1, Q3 at the beginning of session 2, and Q4 at the end of session 2.

#### Comparison of Skills Acquisition and Retention

Two independent blinded raters (an anesthetist and a human factors expert specialized in health sciences) evaluated the technical and nontechnical skills retrospectively by analyzing the video recordings.

Technical skills were assessed by the Neonatal Resuscitation Performance Evaluation (NRPE) scoring system [30], with 20 points for each scenario (eg, checked the material, dried the newborn, and initiated mask ventilation). Nontechnical skills were assessed by the Anaesthetists’ Non-Technical Skills (ANTS) [31] observation system, including four categories: situation awareness, task management, team work, and decision making (eg, prioritizing, coordinating activities with team members, gathering information, and selecting options). ANTS is a validated tool used to assess nontechnical skills in various situations, ranging from emergencies for medical students [32] to neonatal resuscitation for midwives (with a specific modified ANTS version) [33]. ANTS scores were recorded as the overall category scores on a scale of 1-4 from poor performance to good performance and on the 16-point global score as per ANTS system. Interrater reliability calculations were performed for both evaluations, with a good agreement between the two raters (κ=0.66; *P*=.01).

#### Comparison of Self-Efficacy Evaluation

The self-efficacy question assessed midwives’ perception on their own performance: “How much are you confident in your capability to organize and execute a neonatal resuscitation?” using a 6-point Likert scale ranging from “not at all confident” (scored as 0) to “very confident” (scored as 5) [34]. It was assessed at the end of each session.

### Statistical Analysis

Data are presented as median (IQR) for continuous data given the small sample size. Agreement between raters for the ANTS and NRPE scores was evaluated using percent agreement and corresponding Cohen kappa coefficient (inter-rater agreement). Comparisons between groups were performed using the Mann-Whitney *U* test for independent samples. All tests were two-tailed, and statistical significance was considered at *P*<.05. Statistical analyses were performed using SPSS 25.0 software (IBM Corp).

## Results

The study included 28 participants; 14 were randomly assigned to the control group and 14, to the debriefing group. The participants were fourth-year students of a 5-year curriculum of midwifery in France. A majority (27/28) were women. The median (IQR) age was 22 (21-22) years. Five participants had previously followed high-fidelity simulation training, one had followed screen-based simulation training, but none had followed any training on neonatal resuscitation. No participant had witnessed or participated in a real neonatal resuscitation in 2 months prior to the study or had received any training.

### Comparison of Knowledge Acquisition and Retention

At baseline, the control group (median 14.5; IQR 12.5-16) had better results than the debriefing group (median 12.5; IQR 11-13.75) (*P*=.05). This difference disappeared over time. There is no difference between the groups during session 1 and session 2. Results are presented in Table 1.

**Table 1:** Comparison of the knowledge questionnaires of the control and debriefing groups.

Questionnaires of the groups		Median (IQR)	*U*	*P* value
**Q1 baseline**			**56**	**.05**
	Debriefing group	12.5 (11-13.75)		
	Control group	14.5 (12.5-16)		
**Q2 at the end of session 1**			**66**	**.15**
	Debriefing group	13 (12.25-14)		
	Control group	14 (12.25-16)		
**Q3 in session 2**			**82**	**.47**
	Debriefing group	14.5 (13-16)		
	Control group	14 (13.25-15)		
**Q4 at the end of session 2**			**83**	**.48**
	Debriefing group	14 (13.25-14.75)		
	Control group	14 (12.5-15.75)		

### Comparison of Skills Acquisition and Retention

#### Technical Skills Assessment Through the NRPE

No significant difference in the NRPE scores was observed during the experimentation (Table 2).

**Table 2:** Comparison of the nontechnical skills, technical skills, and self-efficacy evaluation between the debriefing and control groups.

		Baseline				Session 1				Session 2			
		Debriefing	Control	*U*	*P* value	Debriefing	Control	*U*	*P* value	Debriefing	Control	*U*	*P* value
**ANTS^a^ score (total=16 points), median (IQR)**		**8 (6.1-9.8)**	**6.75 (5.6-7.9)**	**78.5**	**.38**	**13.25 (11.1-14.4)**	**9 (6.6-11.4)**	**47.5**	**.02**	**10 (9.3-13.9)**	**7.75 (6.5-12)**	**60.5**	**.08**
	Task management (total=4 points)	2 (1.37-2.5)	1.5 (1-2)	70	.18	3 (2.5-3.62)	2 (1.5-3)	43	.10	2 (2-3.62)	2 (1-3)	78	.34
	Team work (total=4 points)	2 (1.37-3)	2 (1-2.5)	80	.39	3.75 (2.87-4)	2.25 (1.87-3.62)	54	.04	4 (3-4)	3.25 (1.87-4)	65.5	.11
	Situation awareness (total=4 points)	2 (1.5-2.5)	2 (1.37-2.5)	87	.60	3.25 (2.87-3.62)	2.25 (1.5-3.62)	60	.08	2.25 (1.87-3.62)	1.5 (1-3)	65.5	.13
	Decision making (total=4 points)	1.5 (1-2.5)	1.5 (1-2.5)	94	.85	3 (2.37-3.5)	2.25 (1.37-3.5)	61	.08	2 (1.87-3.5)	2 (1-3)	70	.19
NRPE^b^ score (total=20 points), median (IQR)		10 (7.3-12.3)	10 (7.3-12.3)	94.5	.87	9.2 (7.7-13.5)	10 (9.2-13.1)	87.5	.62	10.5 (8-12.8)	10.5 (7.5-12)	97	.96
Self-efficacy (total=5 points), median (IQR)		N/A	N/A	N/A	N/A	2 (1-2)	2 (1-2)	92	.76	3 (2-3)	2 (1-2)	52	.02

^a^ANTS: Anaesthetists’ Non-Technical Skills.

^b^NRPE: Neonatal Resuscitation Performance Evaluation.

#### Nontechnical Skills Assessment Through the ANTS

A significant difference was observed between the two groups for session 1 (*U*=47.5; *P*=.02) and remained higher in favor of the debriefing group during session 2 (*U*=60.5; *P*=.08), while no difference was found in the baseline evaluation (scenario 1). The results (including the subcategories analysis) are presented in Table 2.

### Comparison of Self-Efficacy Evaluation

A significant difference was found between the groups for session 2, with an improved self-efficacy score for the debriefing group (Table 2).

## Discussion

### Major Findings

This study highlights the benefit of a computer debriefing compared to no debriefing on nontechnical skills acquisition, self-efficacy, and knowledge after a screen-based simulation of neonatal resuscitation. Our hypothesis that the debriefing group would obtain better scores than the control group in the different evaluations is validated for knowledge, nontechnical skills, and self-efficacy.

The major interest of debriefing after a simulation session has already been extensively demonstrated. The review by Cheng et al [15], including 108 studies comparing debriefing and no debriefing, found positive effects of debriefing on every knowledge and skills outcomes. From debriefing comes a major part of the theoretical benefit of screen-based simulation for training, contributing to meaningful connections between the learning experience and the real world [21]. However, our study was the first to compare computer debriefing and no debriefing and to analyze their impact on knowledge, technical skills, and nontechnical skills after a screen-based simulation.

Concerning knowledge evaluation, participants of the control group had better baseline knowledge of neonatal resuscitation than the debriefing group. Our results showed an improvement in the debriefing group’s score from the baseline level. The differences between the groups disappeared at the end of sessions, reflecting a positive effect of debriefing.

Usually, personalized debriefing after screen-based simulation addresses only technical skills. Data collected from the simulation are given back in the form of an automated feedback at the end of the scenario [21]. We found no evolution for the technical skills in our study. However, the increasing difficulty of the scenarios was designed to minimize the repetition effect on performance, as repeating the same scenario increases the participants’ skills more than varying the scenarios [35]. This could mask the effect of the debriefing itself since the required technical skills evolved with scenarios.

In this study, we added a self-debriefing of nontechnical skills after the screen-based simulation of a neonatal resuscitation. In a review on screen-based simulation for medical education and surgical skills training, Graafland et al [36] highlighted the interest of a screen-based simulation to train nontechnical skills. Furthermore, in a review of debriefing techniques after nontechnical skills simulation training, performance seemed to improve equally with various methods of debriefing: skilled facilitator, novice instructor using a script, and self-led debrief or multimedia debriefing [37]. Our results confirm the possibility and benefit of a self-debriefing of nontechnical skills following a screen-based simulation to improve learning.

The second major finding of this study is the effect of computer debriefing on retention 2 months after the initial training. The debriefing group showed a better self-efficacy assessment than the control group. Their ANTS performance remained higher than that of the control group. The role of debriefing on retention of learning was already underlined in some studies [25,38]. Few studies assessed the retention of learning after screen-based simulation training. Their results were rather positive when evaluated up to 1 month after simulation [38] but less effective than traditional learning methods when evaluated after 6 months [39]. Our positives results are encouraging and emphasize the role of the debriefing in retention of learning even though further studies are needed to confirm a longer-term effect.

### Limitations of the Study

First, this study compared the effect of a computer debriefing with the effect of the absence of debriefing. Our objective was to stay in a virtual environment without the need for an instructor. As debriefing is a major component of simulation training, participants from the control group had access to the complete debriefing at the end of session 2. Therefore, this study only assessed the efficacy of a computer debriefing but not the superiority over other debriefing methods.

Second, the timing of the debriefing was not standardized or assessed. Participants from the debriefing group had an unlimited amount of time to consult the debriefing. This was not the case for the control group. Perhaps, a free time period should have also been proposed to the control group to offer the possibility for a spontaneous reflective process.

Third, the nontechnical skills assessment was performed with the ANTS scoring tool, which was not originally developed and validated for the studied population. The lack of published data on the use of the ANTS scores for midwives is a limitation.

### Conclusion

Computer debriefing seems to improve nontechnical skills and self-efficacy estimation when compared to the absence of debriefing during a screen-based simulation. It also allows a progression of learner’s knowledge. This study supports the benefit of debriefing (including a computer debriefing) in screen-based simulation.

## Abbreviations

ANTS: Anaesthetists’ Non-Technical Skills
CERAR: Comité Ethique sur la Recherche en Anesthésie Réanimation
ILCOR: International Liaison Committee on Resuscitation
NRP: Neonatal Resuscitation Program
NRPE: Neonatal Resuscitation Performance Evaluation
